# Danhong injection in the treatment of idiopathic pulmonary fibrosis: A protocol for systematic review and meta-analysis

**DOI:** 10.1097/MD.0000000000022016

**Published:** 2020-09-11

**Authors:** Yanqiu Lan, Dezhi Wu, Yunrui Jin, Min Shui, Xianjun Fan

**Affiliations:** a Department of Oncology, Army Medical Center of PLA, Chongqing; b Department of Anesthesia, Kaili City First People's Hospital, Kaili; c Department of Oncology, Chongqing General Hospital; d Department of Anesthesia, Army Medical Center of PLA, Chongqing, China.

**Keywords:** danhong injection, idiopathic pulmonary fibrosis, meta-analysis, protocol, systematic review

## Abstract

**Background:**

Many studies have reported that the effects of danhong injection on idiopathic pulmonary fibrosis. However, its effects are still not well understood. The aim of this study is to assess the effects of danhong injection in the management of idiopathic pulmonary fibrosis.

**Methods:**

Electronic databases such as PubMed, MEDLINE, EMbase, Web of Science, Cochrane Library, China National Knowledge Infrastructure, WanFang, the Chongqing VIP Chinese Science and Technology Periodical Database, and China biomedical literature database will be searched without limitations of language and geographical location. Two researchers will independently conduct research selection, data extraction, and research quality assessment. The RevMan 5.3 software and Stata 14.0 software are used for statistical analysis.

**Results:**

This study will provide high-quality comprehensive evidence for the effectiveness and safety of danhong injection in the treatment of idiopathic pulmonary fibrosis.

**Conclusions:**

The results obtained from this study will define the basis for the effectiveness and safety of danhong injection in the treatment of idiopathic pulmonary fibrosis.

## 1 Introduction

Idiopathic pulmonary fibrosis is a serious, progressive, and even fatal disease, and there is no exact and effective cure for this ailment at present.^[1]^ Respiratory viral pneumonia such as coronavirus disease 2019 (COVID-19), especially in severe cases, is more likely to progress into pulmonary fibrosis.^[2]^ Currently, there is a lack of specific drugs in the treatment of idiopathic pulmonary fibrosis.^[3]^ Early corticosteroids and other drugs have not been recommended for clinical application because of their poor efficacy and serious side effects.^[4]^ Even though the new drugs pyrifenidone and nidanib are able to achieve symptomatic relief and improve the rate of deterioration of the pulmonary function to some extent, their overall curative effect is still insufficient.^[5,6]^ Moreover, these drugs have been associated with way too many adverse reactions and their economic profile is poor.^[7,8]^ Therefore, there is still an urgent need to come up with more effective drugs.

In recent years, traditional Chinese medicine has provided new ideas and methods for the treatment of idiopathic pulmonary fibrosis.^[9–11]^ Danhong injection is a traditional Chinese medicine injection derived from salvia miltiorrhiza and carthamus tinctorius via modern technology.^[12]^ It is capable of alleviating the clinical symptoms and pulmonary function of patients with idiopathic pulmonary fibrosis without generating any obvious adverse reaction.^[13]^ To further evaluate the efficacy and safety of danhong injection in the management of patients with Idiopathic pulmonary fibrosis, a meta-analysis of related randomized controlled trials (RCTs) was conducted.

## 2 Methods

### 2.1 Study registration

This study was registered through PROSPERO (PROSPERO Registration number: CRD42020189325). We organized this study based on the Preferred Reporting Items for Systematic Reviews and Meta-Analyses Protocols guidelines.^[14]^

### 2.2 Ethics

Ethical approval is not required as there is no patient recruitment and personal information collection, and the data included in our study were all extracted from published literature.

### 2.3 Inclusion criteria for study selection

#### 2.3.1 Type of studies

We only selected RCTs focusing on the effect of danhong injection in the treatment of idiopathic pulmonary fibrosis, regardless of the language, and publication status restrictions.

#### 2.3.2 Type of participants

RCTs including patients diagnosed with idiopathic pulmonary fibrosis were enrolled, irrespective of the nationality, race, age, gender, and source of cases.

#### 2.3.3 Type of interventions

The control group was treated with conventional western medicine treatment modalities (e.g., pifenidone, nidanib, prednisone, N-acetylcysteine, etc). The treatment group received both danhong injection and western medicine treatment regimens.

#### 2.3.4 Type of outcome measures

The main outcome measure was the clinical effective rate. The clinical effective rate was determined according to the clinical efficacy criteria in the guidelines for diagnosis and treatment of idiopathic Pulmonary (Interstitial) Fibrosis (draft).

The secondary outcomes: Oxygen tension (PaO2); Carbon monoxide diffusing capacity (DLCO); Transforming growth factor-β1(TGF-β1); Hyaluronic acid (HA); Laminin (LN); Type III procollagen; Forced vital capacity in 1 second/forced vital capacity ratio; Type III collagen; Blood urea nitrogen; Adverse reactions.

### 2.4 Exclusion criteria

1. Incomplete data or misrepresentation of data reports.
2. Repeated publication of documents.
3. Case reports, reviews, etc.
4. Inability to obtain original documents.

### 2.5 Search strategy

A comprehensive search of electronic databases such as PubMed, MEDLINE, EMbase, Web of Science, Cochrane Library, China National Knowledge Infrastructure, WanFang, the Chongqing VIP Chinese Science and Technology Periodical Database, and China biomedical literature database was effected to collect RCTs on the integration of danhong injection in the treatment of idiopathic pulmonary fibrosis. We also skimmed other resources for relevant articles. All document sources were not restricted by language and publication status. The search strategy performed for PubMed is illustrated in Table 1. Search of other databases was also performed similarly to this search strategy.

**Table 1:**
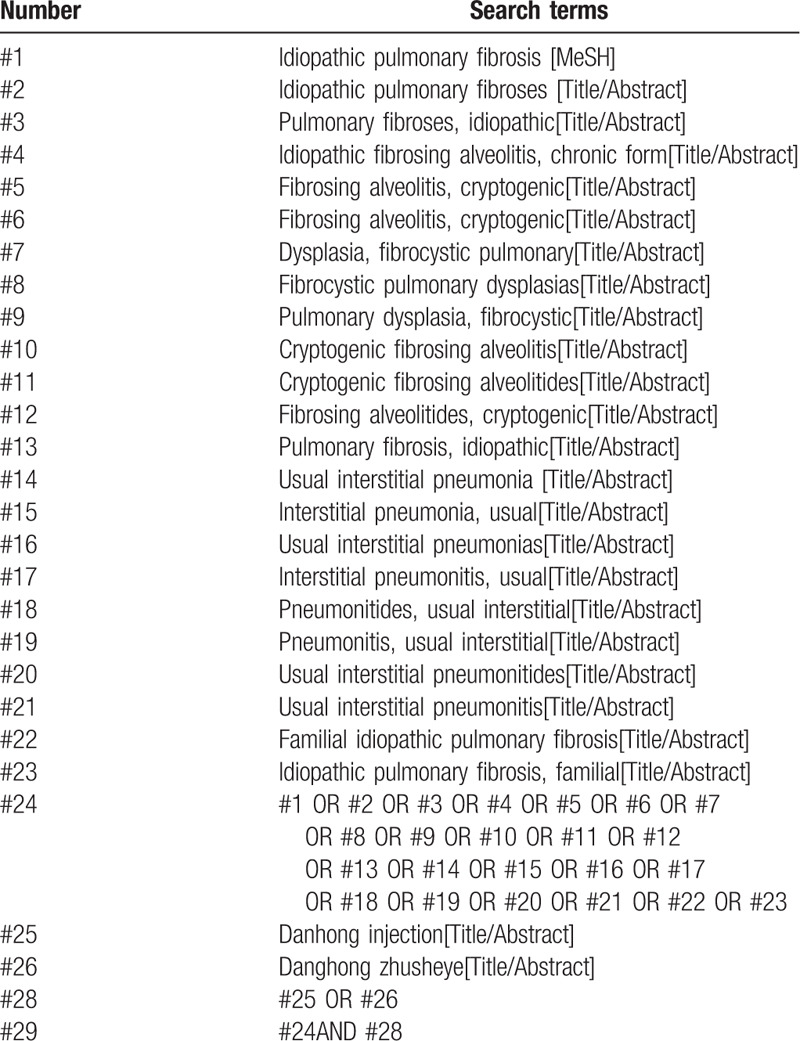
PubMed search strategy.

### 2.6 Data extraction

**Figure 1:**
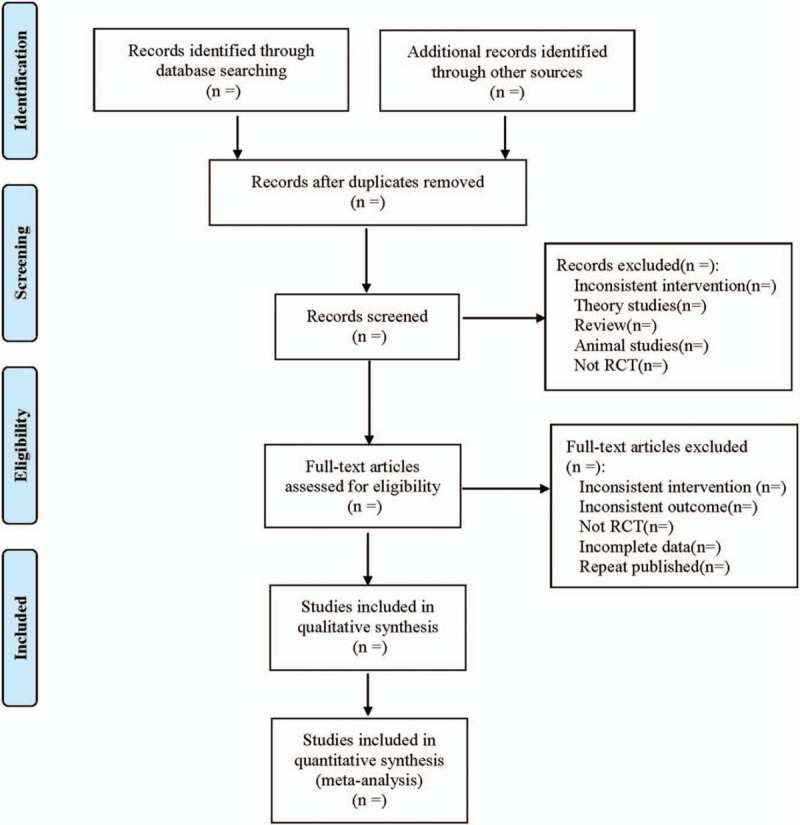
Flow diagram.

Two reviewers independently screened the literature, extracted data, and cross-checked the information. In case of disagreement, a third party was consulted to assist in the judgment, or we tried to contact the author to supplement the missing piece of information. When selecting documents, we first read the title and abstract, and after excluding obviously irrelevant documents, we read the full text to determine whether or not it should be included. The data extracted mainly comprises: Basic information of the included literature, such as the authors, date of publication, country, sample size, age, doses administered, content and duration, intervention time, intervention details, etc; Specific details of the intervention measures, including the usage of danhong injection, dosage administered, etc; Baseline characteristics of the included subjects; Risk of bias in the key elements of evaluation; The prognostic indicators and data measurement, such as the clinical effective rate, PaO2, DLCO, TGF- β1, HA, LN, etc. The literature selection process is displayed in Figure 1.

### 2.7 Risk of bias assessment

Two researchers used the RCT bias risk evaluation tool in the Cochrane System Review Manual 5.1.0 to evaluate the bias risk of the included RCT and cross-check the results. The main items consist of: randomization plan; group concealment; blinding method; incomplete data reporting; selective outcome report; other sources of bias. Each item was evaluated as either “high,” “low,” or “unclear.”

### 2.8 Statistical analysis

#### 2.8.1 Data synthesis

The RevMan 5.3 software (The Cochrane Collaboration, Software Update, Oxford, UK) and Stata 14 software (STATA Corporation, College Station, TX) were used for statistical analysis. For dichotomous variables, odds ratio were implemented for statistical analysis. Meanwhile, for continuous variables, standardized mean difference was selected with different tools or units of measurement, and all the above were represented by effect value and 95% confidence intervals. If the statistical heterogeneity between the results of each study does not exist or is negligible (*I*^*2*^ < 50%, *P* > .1), the fixed-effects model is used for meta-analysis; on the other hand, if there is large statistical heterogeneity detected between the results of the studies (*I*^*2*^ ≥50%, *P* < .1), then we further analyzed the source of heterogeneity. After excluding the influence of obvious etiologies of clinical heterogeneity, the random effects model was used for meta-analysis. The level of meta-analysis was set to α = 0.05. Obvious clinical heterogeneity was solved by subgroup analysis or sensitivity analysis, or only descriptive analysis.

#### 2.8.2 Dealing with missing data

If the relevant data in the literature was incomplete, the first author or corresponding author would be contacted by email or phone to obtain the missing piece of information. If the missing data still could not be obtained through the aforementioned method, we would attempt to synthesize the available data in the preliminary analysis. In addition, a sensitivity analysis would be used to gauge the potential impact of the missing data on the overall results of the study.

#### 2.8.3 Subgroup analysis

The subgroup analysis was based on danhong injection dose, course of treatment, and sample size.

#### 2.8.4 Sensitivity analysis

To test the stability of the meta-analysis results of indicators, a one-by-one elimination method was adopted for sensitivity analysis.

#### 2.8.5 Reporting bias

If the number of included study was ≥10, a funnel plot was used to qualitatively detect publication bias.^[15]^ Egger and Begg tests were used to quantitatively assess any potential publication bias.

## 3 Discussion

Over the past couple of years, studies have demonstrated that idiopathic pulmonary fibrosis is involved in a variety of molecular pathways, including pro-inflammatory cascade, intracellular signal transduction, myofibroblast activation, and so on.^[16–18]^ So far, there is no satisfactory treatment for pulmonary fibrosis.^[19,20]^ Commonly used drugs include glucocorticoids, immunosuppressants/cytotoxic drugs, and antifibrotic agents.^[21]^ Danhong injection is principally composed of salvia miltiorrhiza and safflower extracts, and it has been demonstrated to improve the degree of pulmonary fibrosis.^[13]^ To obtain a thorough picture of the clinical effect of danhong injection as adjuvant and alternative therapy in the management of idiopathic pulmonary fibrosis, we conducted a meta-analysis. This article will provide comprehensive and high-quality evidence for the integration of Danhong injection in the treatment of idiopathic pulmonary fibrosis.

As could be expected, our meta-analysis may have some limitations. First and foremost, including both Chinese and English research literatures may increase the level of bias. Secondly, the diversity of race, age, drug dosage, and treatment course resulted in higher clinical and statistical heterogeneity. In summary, this study will help determine the effectiveness and safety of danhong injection in the treatment pf idiopathic pulmonary fibrosis. We genuinely hope that this study can supply higher-quality evidence for the effectiveness and safety of danhong injection in the management of idiopathic pulmonary fibrosis.

## Author contributions

**Data collection:** MS and XF

**Funding acquisition:** XF

**Resources:** YL

**Software:** DW

**Supervision:** YJ

**Writing – original draft:** YL and DW

**Writing – review & editing:** MS and XF
